# Proteasome inhibitor MG-132 induces MCPIP1 expression

**DOI:** 10.1111/febs.12264

**Published:** 2013-05-07

**Authors:** Lukasz Skalniak, Aleksander Koj, Jolanta Jura

**Affiliations:** 1 Department of General Biochemistry, Faculty of Biochemistry, Biophysics and Biotechnology, Jagiellonian UniversityKrakow, Poland; 2 Department of Cell Biochemistry, Faculty of Biochemistry, Biophysics and Biotechnology, Jagiellonian UniversityKrakow, Poland

**Keywords:** apoptosis, MCPIP1, MG-132, proteasome, ZC3H12A

## Abstract

The proteasome is a protein complex responsible for the degradation of polyubiquitin-tagged proteins. Besides the removal of target proteins, the proteasome also participates in the regulation of gene transcription in both proteolytic and non-proteolytic fashion. In this study the effect of proteasome inhibition on the basal expression of monocyte chemotactic protein-1 induced protein 1 (MCPIP1) was examined. Treatment of HepG2 or HeLa cells with proteasome inhibitor MG-132 resulted in a significant increase of MCPIP1 expression, both at mRNA and protein level. Interestingly, MG-132 did not alter MCPIP1 stability. Instead, the observed protein increase was blocked by actinomycin D, suggesting the involvement of *de novo* mRNA synthesis in the increase of MCPIP1 protein following MG-132 treatment. Using several inhibitors we determined the participation of extracellular-signal-regulated kinase 1/2 and p38 kinases in MCPIP1 upregulation by MG-132. Our findings show for the first time the impact of proteasome inhibition on MCPIP1 protein expression by modulation of the activity of intracellular signaling pathways. Overexpression of MCPIP1-*myc* protein decreased the viability of HeLa cells but not HepG2 cells, which correlates with the increased susceptibility of HeLa cells to MG-132 toxicity. Notably, both MG-132 treatment and MCPIP1-*myc* overexpression led to the activation of apoptosis, as revealed by the induction of caspases 3/7 in both types of cell lines. This suggests the involvement of MCPIP1 upregulation in toxic properties of proteasome inhibition, which is an acknowledged approach to the treatment of several cancer types.

## Introduction

The ubiquitin-proteasome system is crucial for maintaining cell homeostasis with respect to the regulation of protein recycling and quality control for newly synthesized proteins. Inhibition of the proteasome by pharmacological inhibitors such as bortezomib 1 or carfilzomib 2 has become a successful strategy of choice in relapsed and refractory multiple myeloma and mantle cell lymphoma 3–5. Proteasome inhibition results in cell death by apoptosis due to the induction of endoplasmic reticulum stress and generation of reactive oxygen species (ROS) 6. Additional ROS-dependent and independent mechanisms have been postulated to be involved, including stabilization of cell cycle regulators and pro-apoptotic factors, inhibition of nuclear factor-κB (NF-κB), inhibition of protein translation and sensitization to ligand-induced apoptosis 7.

Monocyte chemotactic protein-1 induced protein 1 (MCPIP1) is a multifunctional regulatory protein (reviewed in 8). The protein has been shown to be involved in negative regulation of macrophage activation 9,10 and differentiation of several cell types, including pre-adipocytes 11, neuroprogenitor cells 12 and osteoclast precursors 13. Moreover, MCPIP1 has been postulated to induce apoptosis and autophagy 14–16. The underlying mechanisms of MCPIP1 functioning are very complex. The protein was initially characterized as a transcription factor 14, then as a negative regulator of NF-κB 9,17 and finally described as a ribonuclease (RNase) directly responsible for the degradation of transcripts encoding interleukin 6 (IL-6), IL-1β, IL-2 and IL-12b as well as pri-miRNA 18–21.

Recently it has been shown that MCPIP1 undergoes rapid degradation following stimulation of HeLa cells with IL-1β 22. It was proposed that such a removal of MCPIP1 protein allows for the expression of IL-6 transcript, one of the direct targets of MCPIP1 RNase activity. The authors defined two serine residues in positions 435 and 439 of the murine MCPIP1 to be phosphorylated in response to IL-1β treatment as a signal for the following protein ubiquitination and proteasomal degradation 22.

In this study we addressed the issue of the regulation of basal expression of MCPIP1 by proteasome inhibition. Treatment of HepG2 and HeLa cells with the inhibitor MG-132 significantly increased the level of MCPIP1 mRNA and resulted in a long-term increase of MCPIP1 protein quantity. Moreover, using an overexpression approach we show here that MCPIP1 protein increase may be partially responsible for the toxic effects of pharmacological proteasome inhibitors, such as MG-132. Our findings show for the first time the correlation between proteasome-targeted cancer therapy and the expression and toxicity of MCPIP1 in inhibitor-treated cells.

## Results

### Proteasome inhibitor MG-132 increases the expression of MCPIP1

Mouse MCPIP1 was recently reported to be degraded by a ubiquitin-proteasome system in response to IL-1β 22. In order to explore the engagement of the proteasome in the control of MCPIP1 expression we used proteasome inhibitor MG-132. Besides proteasome inhibition, MG-132 has also been reported to inhibit calpains. Therefore, to minimize toxic and side effects of MG-132, a low dose of the inhibitor was used (1 μm). Such a low MG-132 concentration is below the IC_50_ of calpain inhibition *in vitro* and far below the reported IC_50_ of calpain inhibition measured in a cell-based assay 23.

**FIG 1:**
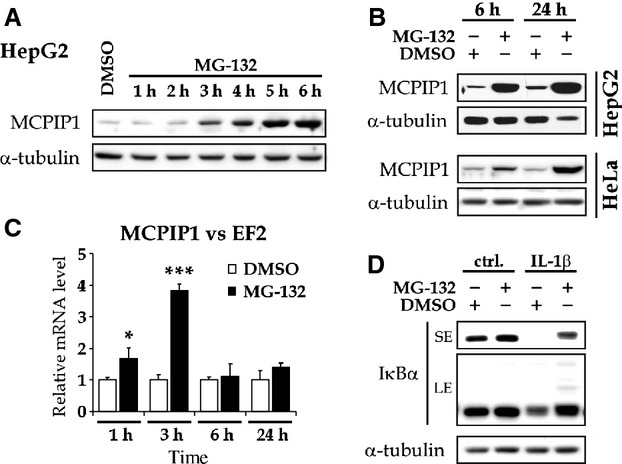
Proteasome inhibitor MG-132 increases the expression of MCPIP1. (A), (B) HepG2 or HeLa cells (as indicated) were treated with 1 μm MG-132 or DMSO for the indicated time periods. Protein extracts were subjected to western blotting with MCPIP1- and α-tubulin-specific antibodies. (C) HepG2 cells were treated with 1 μm MG-132 or DMSO for the indicated time periods. Total RNA was isolated and real-time PCR was performed. MCPIP1 transcript level was normalized to EF2 transcript. The graph shows means ± SE from three independent experiments, presented as fold change versus DMSO-treated control at each time point. For the statistics the *t*-test was performed: ^*^P < 0.05, ^***^P < 0.001 versus control. (D) HepG2 cells were pretreated with 1 μm MG-132 or DMSO for 1 h and subjected to 5 min stimulation with 10 ng·mL^−1^ IL-1β. Protein extracts were subjected to western blotting with IκBα- and α-tubulin-specific antibodies (SE, short exposure; LE, long exposure). Blots A, B and D are representative from three independent experiments.

MG-132 remarkably increased the expression of MCPIP1 protein in HepG2 cells (Fig. 1A). The level of MCPIP1 protein increased time-dependently starting from the third hour after MG-132 treatment (Fig. 1A). The increase was not observed at early time points (1 and 2 h following MG-132 administration). A similar increase of MCPIP1 after MG-132 was observed in the HeLa cell line following 6 h of treatment (Fig. 1B). The elevated MCPIP1 protein amount was prolonged and even more evident 24 h after treatment in both HepG2 and HeLa cells (Fig. 1B).

Using real-time PCR we checked the influence of MG-132 on the MCPIP1 transcript. HepG2 cells were stimulated with 1 μm MG-132 for 1, 3, 6 and 24 h. The treatment with MG-132 for 3 h resulted in an almost four-fold increase of the level of MCPIP1 mRNA (Fig. 1C). The observed elevated mRNA level was temporary and returned to the basal level at the later tested time points.

The inhibition of proteasome by MG-132 at a concentration of 1 μm was verified by analysis of the inhibitor of NF-κB (IκBα) degradation. MG-132 was administered for 1 h, after which HepG2 cells were stimulated with 10 ng·mL^−1^ of IL-1β for 5 min, which resulted in degradation of IκBα (Fig. 1D). This degradation was reduced but not completely blocked when MG-132 was present, suggesting that a weak proteasome activity is maintained in the presence of the low MG-132 dose used (Fig. 1D).

### Increased expression of MCPIP1 following MG-132 requires mRNA synthesis but does not involve protein stabilization

**FIG 2:**
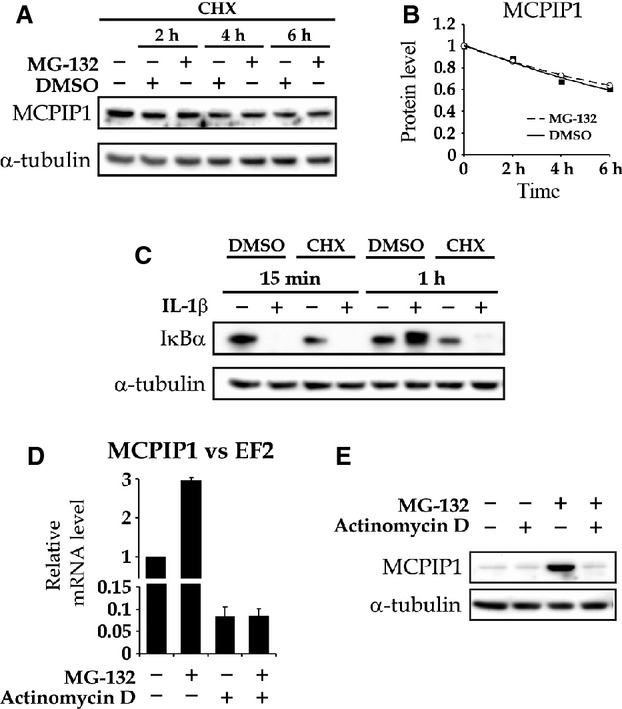
MCPIP1 upregulation by MG-132 requires protein and mRNA *de novo* synthesis. (A) HepG2 cells were pretreated with 5 μg·mL^−1^ cycloheximide (CHX) for 30 min, followed by treatment with 1 μm MG-132 or DMSO for the indicated time periods. Untreated cells served as control. Protein extracts were subjected to western blotting with MCPIP1- and α-tubulin-specific antibodies. (B) Quantification of the blot in (A) presented as an optical density relative to untreated cells. (C) HepG2 cells were pretreated with cycloheximide (CHX) or DMSO for 30 min and stimulated with 10 ng·mL^−1^ IL-1β for 5 min or 1 h. Protein extracts were subjected to western blotting with IκBα- and α-tubulin-specific antibodies. (D), (E) HepG2 cells were pretreated with 5 μg·mL^−1^ actinomycin D for 1 h followed by treatment with 1 μm MG-132 for 3 h for RNA collection (D) or 6 h for protein analysis (E). MCPIP1 transcript was normalized to EF2 transcript. Graph D shows means ± SE from three independent experiments, presented as fold change versus control. Blots A, C and E are representative from three independent experiments.

Recently it was shown that MCPIP1 undergoes proteasomal degradation following stimulation with IL-1β 22. To check if protein stabilization is responsible for the increase of MCPIP1 level upon MG-132 treatment, HepG2 cells were pretreated with cycloheximide for 30 min and then treated with MG-132 for 2, 4 or 6 h. In cycloheximide-treated cells MG-132 failed to induce MCPIP1 expression, suggesting the importance of *de novo* protein synthesis in MCPIP1 upregulation (Fig. 2A). After 6.5 h of cycloheximide treatment the level of MCPIP1 expression had decreased to 60%; however, the presence of MG-132 did not alter the stability of MCPIP1 (Fig. 2A,B).

To test the desired inhibition of protein synthesis by cycloheximide, HepG2 cells were pretreated with cycloheximide and then stimulated with 10 ng·mL^−1^ of IL-1β for 15 min or 1 h. The stimulation with IL-1β led to rapid degradation of IκBα in both dimethylsulfoxide (DMSO)-treated and cycloheximide-treated cells (Fig. 2C). In contrast, IκBα re-synthesis after 1 h of stimulation was observed only in DMSO-treated cells, suggesting successful inhibition of protein synthesis in cycloheximide-treated cells (Fig. 2C).

To verify whether the observed increase of MCPIP1 transcript following MG-132 treatment is necessary for the increase of MCPIP1 at the protein level we blocked *de novo* RNA synthesis with actinomycin D. Actinomycin D was applied 1 h before MG-132 treatment, after which the cells were cultured for 3 h in the presence of MG-132. The presence of actinomycin D decreased the basal amount of MCPIP1 mRNA by 90% and blocked the increase of MCPIP1 mRNA observed following MG-132 administration (Fig. 2D). This was accompanied by the actinomycin D-dependent inhibition of MG-132-mediated MCPIP1 protein increase (Fig. 2E). Actinomycin D had no effect on the basal MCPIP1 protein level (without MG-132, Fig. 2E).

### ERK1/2 and p38 are involved in MCPIP1 upregulation induced by MG-132

**FIG 3:**
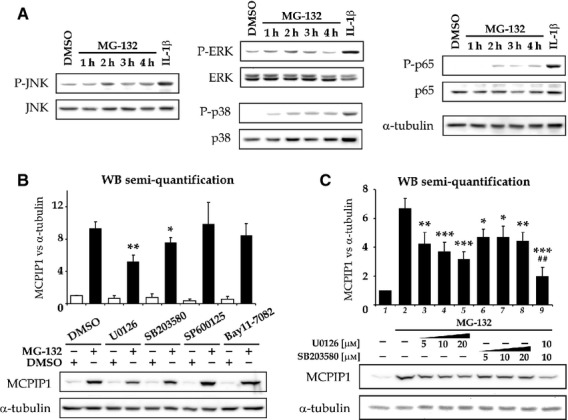
Involvement of signaling pathways in MG-132-triggered MCPIP1 increase. (A) HepG2 cells were treated with 1 μm MG-132 for the indicated time periods or with 10 ng·mL^−1^ of IL-1β for 30 min. Cell lysates were subjected to western blotting with the indicated antibodies. Representative blots from three independent experiments are shown. (B) HepG2 cells were pretreated with 10 μm U0126, 10 μm SB203580, 10 μm SP600125 or 20 μm Bay11-7082 for 30 min and treated with 1 μm MG-132 or DMSO for an additional 6 h. Protein extracts were subjected to western blotting with MCPIP1- and α-tubulin-specific antibodies. The graph represents densitometry quantification of blots and shows means ± SE from three independent experiments. For the statistics the *t*-test was performed: *P < 0.05, **P < 0.01 versus control cells (pretreated with DMSO and treated with MG-132). (C) HepG2 cells were pretreated for 30 min with 5, 10 or 20 μm of U0126, 5, 10 or 20 μm of SB203580 or both inhibitors at a final concentration of 10 μm each, and treated with 1 μm MG-132 or DMSO for an additional 6 h. Protein extracts were subjected to western blotting with MCPIP1- and α-tubulin-specific antibodies. The graph represents densitometry quantification of blots and shows means ± SE from three independent experiments. For the statistics one-way analysis of variance followed by Tukey’s HSD test was used: *P < 0.05, **P < 0.01, ***P < 0.001 versus control cells in lane 2 (pretreated with DMSO and treated with MG-132); ^##^*P* < 0.01 versus cells pretreated with 10 μm SB203580 and treated with MG-132, lane 7.

It was shown before that the inhibition of proteasome activates several intracellular signaling pathways 24. To verify this in our model we treated HepG2 cells with 1 μm of MG-132 for 1, 2, 3 and 4 h and analyzed the phosphorylation status of mitogen-activated protein kinases (MAPKs) and the p65 subunit of NF-κB. Stimulation with IL-1β for 30 min served as a positive control. MG-132 markedly increased the phosphorylation of p38 kinase and p65 protein, starting from the first and second hour of stimulation respectively (Fig. 3A). MG-132 treatment resulted also in a weak and transient activation of extracellular-signal-regulated kinases (ERKs) 1/2 with a maximum after 2 h following MG-132 administration (Fig. 3A). IL-1β treatment resulted in a rapid phosphorylation of all four mentioned proteins (Fig. 3A).

Using four pharmaceutical inhibitors, U0126 for inhibiting MAP kinase kinase (MEK) 1 and 2 (and thus ERK1/2) phosphorylation, SB203580 for p38, SP600125 for c-Jun N-terminal kinases (JNKs) and Bay11-7082 for IκBα phosphorylation and degradation, we elucidated the involvement of the mentioned signaling pathways in MG-132-induced MCPIP1 protein increase. The inhibitors were applied for 1 h before MG-132 treatment. Inhibition of ERK1/2 by 10 μm U0126 and p38 by 10 μm SB203580 significantly blunted the increase of MCPIP1 evoked by MG-132 treatment (Fig. 3B). Other tested inhibitors gave no significant effects at the tested concentrations (10 μm of SP600125 and 20 μm of Bay11-7082). The use of a lower concentration of U0126 (5 μm) and SB203580 also resulted in a significant decrease of MCPIP1 protein, upregulated by MG-132 treatment (Fig. 3C). The use of higher concentrations (20 μm) of each of these inhibitors gave even stronger effects (Fig. 3C). The observed impact of ERK1/2 and p38 inhibition on MCPIP1 expression was additive, since pretreatment of HepG2 cells with a mixture of both inhibitors resulted in a significantly greater decrease of MCPIP1 level (Fig. 3C, lane 9) compared with cells treated with SB203580 alone (Fig. 3C, lane 7). The use of the mixture of these two inhibitors resulted in almost complete inhibition of MG-132-triggered increase of MCPIP1 expression (Fig. 3C, lane 9 versus lane 1, *P* = 0.5867).

### HeLa cells are more susceptible to both MG-132 toxicity and MCPIP1-triggered death than HepG2 cells

Inhibition of the proteasome results in activation of programmed cell death 25. To compare the toxic effects of MG-132 on HepG2 and HeLa cells we treated the cells with MG-132 in concentrations ranging from 0.125 to 8 μm. The induction of apoptosis was verified by the measurement of caspase 3/7 activity and the overall toxicity of MG-132 was estimated by 3-(4,5-dimethylthiazol-2-yl)-2,5-diphenyl-tetrazolium bromide (MTT) assay. Both tests were performed 24 h after MG-132 treatment.

**FIG 4:**
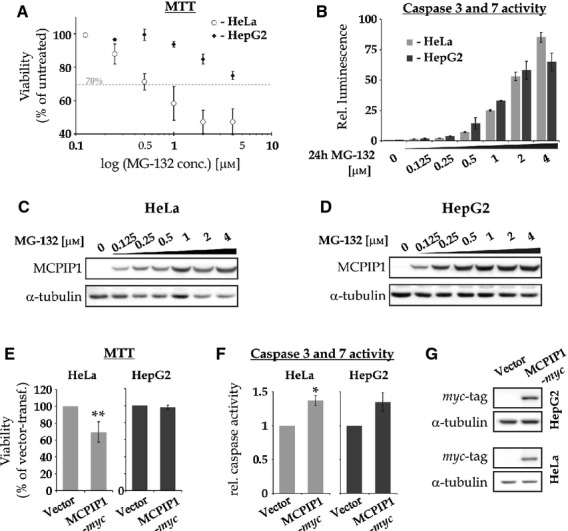
MG-132 and MCPIP1 toxicity in HeLa and HepG2 cells. (A), (B) HepG2 and HeLa cells were treated with different concentrations of MG-132 for 24 h. For the determination of cell viability the MTT test was performed (A) and for verification of apoptosis induction activity of caspase 3 and 7 was measured (B). The graphs show means ± SE from three independent experiments, each performed in five replicates (MTT) or duplicates (caspase activity), presented as a percentage of control (A) or as fold change versus control (B). (C), (D) Western blotting verification of MCPIP1 induction in protein extracts used for caspase 3 and 7 activity measurements (B). Blots are representative from three independent experiments. (E), (F) HepG2 and HeLa cells were transfected with MCPIP1-*myc* coding or an empty vector. Two days following transfection the MTT test (E) and the caspase 3 and 7 activity test (F) were performed. The graphs show means ± SE from four independent experiments, each performed in five replicates (MTT) or duplicates (caspase activity), presented as a percentage of control (E) or as fold change versus control (F). Overexpression of MCPIP1-*myc* in HepG2 and HeLa cells was verified by western blot (G).

The viability of HeLa cells was reduced by about 10% already for the second lowest concentration of MG-132 used (0.25 μm) (Fig. 4A). A concentration of 0.5 μm of MG-132 reduced HeLa viability to 70%. For HepG2 cells a similar toxicity of MG-132 was achieved at a concentration of 8 μm, whilst 0.5 μm did not affect the viability of these cells (Fig. 4A). As an indicator of apoptosis-related processes caspase 3 and 7 activity was measured. MG-132 remarkably and dose-dependently induced caspase 3 and 7 in both HeLa and HepG2 cells (Fig. 4B). Surprisingly, the fold induction of caspase activity was comparable for both cell lines at the same MG-132 concentrations (Fig. 4B).

The expression of MCPIP1 in cells treated with diverse doses of MG-132 was verified by western blotting (Fig. 4C,D). In both cell lines the MCPIP1 level was raised in a dose-dependent manner.

To verify the influence of elevated MCPIP1 expression on the viability of HeLa and HepG2 cell lines, the cells were transfected with vectors encoding MCPIP1-*myc* fusion protein or an empty vector as control. The MTT test and caspase 3/7 activity assay were performed 48 h following transfection. Overexpression of MCPIP1-*myc* protein reduced the viability of HeLa cells by 30% (Fig. 4E), while HepG2 cell viability was not affected. Forced MCPIP1-*myc* expression resulted also in a weak but significant activation of caspases 3 and 7 in both cell lines tested (Fig. 4F). The overexpression of MCPIP1-*myc* protein was verified by western blotting (Fig. 4G).

## Discussion

The ubiquitin-proteasome system is the most important pathway for the quality control of newly synthesized proteins and the removal of intracellular proteins. In some cases the proteasome additionally functions as a processing unit, relieving functional proteins from their precursors by limited proteolysis 26 (proposed mechanisms are discussed in 27). During the last few years the proteasome has also been linked to transcriptional regulation of gene expression. The classical examples of this action of the proteasome include ubiquitin-dependent removal of transcription factors (e.g. p53) or their negative regulators (e.g. IκBα), modulated in response to adequate signals. Recently, however, increasing evidence shows that the proteasome also provides non-proteolytic functions involved in multiple aspects of transcription-related processes (reviewed in 28).

According to the data collected by the Human Protein Atlas project 29 and our data 19 MCPIP1 (*ZC3H12A*) expression is weak or moderate in most of the analyzed cell types and organs. Moderate staining was reported especially for epithelial cells and several other cell types, including skin and lung-residing macrophages, while strong staining was observed in adrenal gland and kidney. Considering its functions the low expression level of MCPIP1 protein seems to be important for cell functioning and survival. Since MCPIP1 is a regulatory protein it also requires mechanisms of a fine regulation of its expression and activity.

In our study we show that proteasome inhibition by a low dose of MG-132 results in a tremendous increase of the expression of MCPIP1 protein in both HepG2 and HeLa cell lines (Fig. 1A,B). As revealed by the study with actinomycin D and cycloheximide, the increase of MCPIP1 expression by the inhibition of proteasome was a consequence of the elevation of mRNA level and requires new protein synthesis. Inhibition of transcription blocked the increase of both MCPIP1 mRNA and protein (Fig. 2D,E). Similarly, inhibition of translation with cycloheximide inhibited MCPIP1 protein increase following MG-132 treatment (Fig. 2A). Interestingly, proteasome inhibitor MG-132 did not result in MCPIP1 protein stabilization (Fig. 2B), which might indicate that either another protease is engaged in the removal of MCPIP1 or, more likely, that the remaining proteasome activity upon inhibition with low dose MG-132 (1 μm) is sufficient for normal MCPIP1 turnover. Indeed, the inhibition of proteasome by 1 μm MG-132 was not complete, as this concentration of proteasome inhibitor did not fully protect IκBα from proteasomal degradation in the presence of IL-1β (Fig. 1D).

The induction of gene transcription following proteasome inhibition has been observed before for several other proteins, e.g. IL-6 30, IL-8 31, heme oxygenase-1 32 or cyclooxygenase-2 24. In these studies the observed induction of gene expression was driven by one of the MAPK pathways, JNK, p38 or ERK1/2, depending on the transcript from the above-mentioned list. The kinases are activated in response to elevated ROS production following proteasome inhibition, since ROS scavengers *N*-acetylcysteine and glutathione prevent kinase phosphorylation 24. Our results support previously published observations that MG-132 treatment leads to the activation of all three MAPKs. Additionally, we show that in the case of MCPIP1 upregulation by MG-132 the ERK1/2 cascade plays the major role with p38 activation of secondary importance (Fig. 3B,C).

The involvement of the ERK1/2 cascade in the transcription of the MCPIP1 coding gene (namely *ZC3H12A*) was proposed earlier in cells stimulated with IL-1β 33. In this study two transcription factors, Elk-1 and SRF, were found to bind to the proximal promoter of this gene following stimulation, in an ERK1/2-dependent manner. Thus the involvement of ERK1/2 in MG-132-induced *ZC3H12A* activation is not surprising. In a previous study we have shown that *ZC3H12A* transcription induction following IL-1β requires also NF-κB activation 17. Short treatment with MG-132 has been shown to inhibit NF-κB activation 34; thus the involvement of NF-κB in rapid *ZC3H12A* transcription following MG-132 administration is not expected. The involvement of p38 in MCPIP1 expression is a novel aspect in the regulation of MCPIP1 expression. p38 was linked to the stabilization of transcripts containing AU-rich regions 35. Recently, p38 was found to be responsible for the stabilization of mRNA encoding nonsteroidal anti-inflammatory drug-activated gene-1 in response to MG-132 in glioblastoma cells 36. Additionally, p38 kinase was postulated to stabilize tristetraprolin, another CCCH-containing protein involved in the regulation of mRNA stability, by protection from proteasomal degradation 37. However, in our study MG-132 provided neither mRNA nor protein stabilization (Fig. 2D,B), suggesting other molecular mechanisms of p38 engagement in MCPIP1 expression.

MCPIP1 was shown to induce ROS production, endoplasmic reticulum stress, apoptosis and autophagy in the rodent cardiomyoblast cell line H9c2 16,38. The role of MCPIP1 in primary cells needs further evaluation, since on one hand the protein was shown to mediate toxic effects of hyperglycemia in rodent cardiomyocytes 38, but on the other hand overexpression of MCPIP1 in myocardium did not activate apoptosis in those cells 39. However, due to the postulated deleterious nature of MCPIP1, the sustained elevated protein level may contribute to the toxic effect of proteasome inhibition by MG-132. As shown in our study, MCPIP1 overexpression reduces the viability of HeLa cells but not HepG2 cells, which are more resistant to the toxic effects of MG-132. Interestingly, in both cell lines a weak but comparable activation of effector caspases (caspases 3 and/or 7) was observed. This suggests that either HepG2 cells are less susceptible to apoptosis caused by caspase activation or the limited viability of HeLa cells upon MCPIP1 overexpression results from additional mechanisms affected by MCPIP1.

Proteasome inhibition was shown to block IκBα degradation and NF-κB activation following IL-1β and tumor necrosis factor α treatment 34,40. On the other hand prolonged inhibition of proteasome with bortezomib or MG-132 resulted in caspase 8 or calpain-dependent degradation of IκBα and activation of NF-κB 41,42. The inhibition of NF-κB by the stable overexpression of IκBα was found to reduce the growth of HeLa cells 43. Since MCPIP1 is known to downregulate NF-κB signaling at both basal and lipopolysaccharide-induced levels 9,17, its toxicity in HeLa cells may result from the inhibition of pro-survival properties of NF-κB. Currently, we are verifying the involvement of NF-κB inhibition in toxic effects of MCPIP1 expression. We are also investigating the mechanisms of reduced toxicity of both MG-132 and MCPIP1 in HepG2 cells in comparison with HeLa cells.

To summarize, previous studies convincingly demonstrate that MCPIP1 serves as a regulator of inflammatory-related processes by its RNase and NF-κB inhibitory properties. Here we present evidence that MCPIP1 additionally seems to be a subtle and balanced inducer of cell death and that its toxic properties vary depending on the type of cells analyzed. These toxic properties of MCPIP1 may contribute to cell death following pharmacological proteasome inhibition; however, the underlying mechanisms of MCPIP1 toxic action require further investigation.

## Materials and methods

### Materials

MG-132 inhibitor was from Tocris Bioscience (Bristol, UK). For the inhibition of signal transduction pathways the following inhibitors were used: MEK1 and MEK2 (thus ERK1/2) inhibitor U0126 (Sigma Aldrich), JNK1/2/3 inhibitor SP600125 (Sigma Aldrich), p38α/β/β2 inhibitor SB203580 (Sigma Aldrich) and IκBα degradation inhibitor (NF-κB inhibitor) BAY11-7082 (Sigma Aldrich). Actinomycin D and cycloheximide were from Sigma Aldrich. Human recombinant IL-1β was purchased from PromoKine. Plastic materials were from BD Falcon.

### Cell culture and treatment, cell transfection

Human hepatocellular liver carcinoma cell line (HepG2) and human epithelial carcinoma cell line (HeLa) were purchased from the ATCC collection. HepG2 cells from passages 95–105 were used. Both cell lines were cultured in a humidified atmosphere at 37 °C and 5% CO_2_ in DMEM containing 1 g·L^−1^ glucose (Lonza, Basel, Switzerland) supplemented with 5% fetal bovine serum (Lonza). For the experiments cells were seeded on cell culture plates (BD Falcon, San Jose, CA, USA). HepG2 cells were seeded on poly-l-lysine-coated plates.

MAPK and NF-κB inhibitors were applied 30 min before MG-132 treatment at the concentrations indicated in the figures. All inhibitors used were dissolved in DMSO; thus DMSO was used as control. MG-132 inhibitor or IL-1β were applied following overnight serum starvation in DMEM supplemented with 0.5% fetal bovine serum. For MCPIP1-*myc* overexpression, cells were transfected with Lipofectamine 2000 (Life Technologies, Carlsbad, CA, USA) according to the manufacturer’s instructions. To reduce the toxicity of Lipofectamine 2000 the medium was changed 4 h following transfection.

### Western blotting

Total cell lysates were prepared using RIPA buffer (25 mm Tris/HCl, pH 7.6, 150 mm NaCl, 1% NP-40, 1% sodium deoxycholate, 0.1% SDS) with protease inhibitor cocktail (Sigma Aldrich) and separated on SDS/PAGE 12% polyacrylamide gel. For phosphorylated protein detection phosphatase inhibitor cocktail (PhosSTOP; Roche, Basel, Switzerland) was added to RIPA buffer. Following the electrotransfer to poly(vinylidene difluoride) membrane (Merck Millipore, Billerica, MA, USA) a blocking agent was applied: 3% milk in Tris-buffered saline containing 0.1% Tween for the Sigma Aldrich, St. Louis, MO, USA (GeneTex, Irvine, CA, USA) and 2% BSA (BioShop, Burlington, Canada) dissolved in Tris-buffered saline containing 0.1% Nonidet for all other antibodies. Membranes were incubated with primary antibody at 4 °C overnight. After four washes, addition of secondary antibodies and an additional four washes, detection was performed using a Luminata Crescendo (Millipore) substrate and MicroChemi chemiluminescence detector (DNR Bio-Imaging Systems, Jerusalem, Israel). The following antibodies and dilutions were used: rabbit monoclonal anti-IκBα (1 : 10 000, cat. ab32518; Abcam, Cambridge, MA, USA), rabbit polyclonal anti-MCPIP1 (1 : 3000, cat. GTX110807; GeneTex), rabbit polyclonal anti-phospho-JNK (1 : 1000, cat. 9251; Cell Signaling, Beverly, MA, USA), rabbit polyclonal anti-JNK (1 : 1000, cat. 9252; Cell Signaling), rabbit polyclonal anti-phospho-p38 (1 : 500, cat. 9211; Cell Signaling, Beverly, MA, USA), rabbit polyclonal anti-p38 (1 : 500, cat. 9212; Cell Signaling), rabbit polyclonal anti-phospho-ERK1/2 (1 : 1000, cat. 9101; Cell Signaling), rabbit polyclonal anti-ERK1/2 (1 : 1000, cat. 9102; Cell Signaling), rabbit monoclonal anti-phospho-p65 (Ser536) (1 : 1000, cat. 3033; Cell Signaling), rabbit polyclonal anti-p65 (0.5 μg·mL^−1^, cat. ab16502; Abcam), rabbit polyclonal anti-Myc-tag (1 : 2000, cat. 2272; Cell Signaling), mouse anti-α-tubulin (1 : 2000, cat. CP06; Calbiochem, Merck Group, Darmstadt, Germany), goat peroxidase-conjugated anti-rabbit (1 : 3000, cat. 7074; Cell Signaling) and goat peroxidase-conjugated anti-mouse (1 : 20 000, cat. 554002; BD Pharminogen, Franklin Lakes, NJ, USA). The densitometry analysis was performed with imagej 1.40 g software 44. All measured values were normalized to α-tubulin expression level.

### Real-time PCR

Total RNA was isolated using a modified Chomczynski–Sacchi method, reverse transcribed and subjected to real-time PCR, as described before 17. The following primers were used for MCPIP1 mRNA: 5′-GGAAGCAGCCGTGTCCCTATG-3′ (forward) and 5′-TCCAGGCTGCACTGCTCACTC-3′ (reverse). Each sample was normalized to reference gene elongation factor 2 (EF2), amplified with the following primers: 5′-GACATCACCAAGGGTGTGCAG-3′ (forward) and 5′-TTCAGCACACTGGCATAGAGGC-3′ (reverse).

### Cell cytotoxicity and apoptosis assays

For MTT cell viability assay cells were seeded on 96-well transparent plates. For the testing of MG-132 toxicity cells were treated with MG-132 for 24 h, following overnight serum starvation. For MCPIP1 overexpression cells were transfected 1 day after passage and cultured for 48 h, with medium change at the fourth hour of transfection. MTT (Sigma) was added for an additional 30 min in a final concentration of 500 ng·mL^−1^. The medium was removed by suction and MTT crystals were dissolved in acidic (40 mm HCl) isopropanol. The absorbance was measured with an Infinite 200 microplate reader (Tecan Group Ltd) at 570 nm with reference wavelength 650 nm.

Caspase 3/7 activity was measured using Caspase-Glo 3/7 Assay (Promega). Cells were seeded on 24-well transparent plates and subjected to a similar treatment as for the MTT test (see above). Protein extracts were isolated with RIPA buffer. Equal amounts of cell lysates (3 μg of proteins) were filled up on white 96-well plates with RIPA buffer to the final volume of 20 μL. Next, 20 μL of Caspase-Glo 3/7 Reagent was added and plates were shaken for 1 min at 300 r.p.m. The plates were kept in the dark at room temperature for 90 min and luminescence was measured with an Infinite M200 microplate reader.

## Abbreviations

DMSO: dimethylsulfoxide
ERK: extracellular-signal-regulated kinase
IL: interleukin
IκB: inhibitor of NF-κB
JNK: c-Jun N-terminal kinase
MAPK: mitogen-activated protein kinase
MCPIP1: monocyte chemotactic protein-1 induced protein 1
MEK: MAP kinase kinase
MTT: 3-(4,5-dimethylthiazol-2-yl)-2,5-diphenyl-tetrazolium bromide
NF-κB: nuclear factor-κB
ROS: reactive oxygen species

## References

[1] Teicher BA, Ara G, Herbst R, Palombella VJ, Adams J (1999). The proteasome inhibitor PS-341 in cancer therapy. Clin Cancer Res.

[2] Kuhn DJ, Chen Q, Voorhees PM, Strader JS, Shenk KD, Sun CM, Demo SD, Bennett MK, van Leeuwen FW, Chanan-Khan AA (2007). Potent activity of carfilzomib, a novel, irreversible inhibitor of the ubiquitin-proteasome pathway, against preclinical models of multiple myeloma. Blood.

[3] Moreau P, Richardson PG, Cavo M, Orlowski RZ, San Miguel JF, Palumbo A, Harousseau JL (2012). Proteasome inhibitors in multiple myeloma: 10 years later. Blood.

[4] Sterz J, von Metzler I, Hahne JC, Lamottke B, Rademacher J, Heider U, Terpos E, Sezer O (2008). The potential of proteasome inhibitors in cancer therapy. Expert Opin Investig Drugs.

[5] Lawasut P, Chauhan D, Laubach J, Hayes C, Fabre C, Maglio M, Mitsiades C, Hideshima T, Anderson KC, Richardson PG (2012). New proteasome inhibitors in myeloma. Curr Hematol Malig Rep.

[6] Fribley A, Wang CY (2006). Proteasome inhibitor induces apoptosis through induction of endoplasmic reticulum stress. Cancer Biol Ther.

[7] Wu WK, Cho CH, Lee CW, Wu K, Fan D, Yu J, Sung JJ (2010). Proteasome inhibition: a new therapeutic strategy to cancer treatment. Cancer Lett.

[8] Jura J, Skalniak L, Koj A (2012). Monocyte chemotactic protein-1-induced protein-1 (MCPIP1) is a novel multifunctional modulator of inflammatory reactions. Biochim Biophys Acta.

[9] Liang J, Wang J, Azfer A, Song W, Tromp G, Kolattukudy PE, Fu M (2008). A novel CCCH-zinc finger protein family regulates proinflammatory activation of macrophages. J Biol Chem.

[10] Liang J, Song W, Tromp G, Kolattukudy PE, Fu M (2008). Genome-wide survey and expression profiling of CCCH-zinc finger family reveals a functional module in macrophage activation. PLoS One.

[11] Younce CW, Azfer A, Kolattukudy PE (2009). MCP-1 (monocyte chemotactic protein-1)-induced protein, a recently identified zinc finger protein, induces adipogenesis in 3T3-L1 pre-adipocytes without peroxisome proliferator-activated receptor gamma. J Biol Chem.

[12] Vrotsos EG, Kolattukudy PE, Sugaya K (2009). MCP-1 involvement in glial differentiation of neuroprogenitor cells through APP signaling. Brain Res Bull.

[13] Wang K, Niu J, Kim H, Kolattukudy PE (2011). Osteoclast precursor differentiation by MCPIP via oxidative stress, endoplasmic reticulum stress, and autophagy. J Mol Cell Biol.

[14] Zhou L, Azfer A, Niu J, Graham S, Choudhury M, Adamski FM, Younce C, Binkley PF, Kolattukudy PE (2006). Monocyte chemoattractant protein-1 induces a novel transcription factor that causes cardiac myocyte apoptosis and ventricular dysfunction. Circ Res.

[15] Qi D, Huang S, Miao R, She ZG, Quinn T, Chang Y, Liu J, Fan D, Chen YE, Fu M (2011). Monocyte chemotactic protein-induced protein 1 (MCPIP1) suppresses stress granule formation and determines apoptosis under stress. J Biol Chem.

[16] Younce CW, Kolattukudy PE (2010). MCP-1 causes cardiomyoblast death via autophagy resulting from ER stress caused by oxidative stress generated by inducing a novel zinc-finger protein, MCPIP. Biochem J.

[17] Skalniak L, Mizgalska D, Zarebski A, Wyrzykowska P, Koj A, Jura J (2009). Regulatory feedback loop between NF-kappaB and MCP-1-induced protein 1 RNase. FEBS J.

[18] Matsushita K, Takeuchi O, Standley DM, Kumagai Y, Kawagoe T, Miyake T, Satoh T, Kato H, Tsujimura T, Nakamura H (2009). Zc3h12a is an RNase essential for controlling immune responses by regulating mRNA decay. Nature.

[19] Mizgalska D, Wegrzyn P, Murzyn K, Kasza A, Koj A, Jura J, Jarzab B, Jura J (2009). Interleukin-1-inducible MCPIP protein has structural and functional properties of RNase and participates in degradation of IL-1beta mRNA. FEBS J.

[20] Suzuki HI, Arase M, Matsuyama H, Choi YL, Ueno T, Mano H, Sugimoto K, Miyazono K (2011). MCPIP1 ribonuclease antagonizes dicer and terminates microRNA biogenesis through precursor microRNA degradation. Mol Cell.

[21] Li M, Cao W, Liu H, Zhang W, Liu X, Cai Z, Guo J, Wang X, Hui Z, Zhang H (2012). MCPIP1 down-regulates IL-2 expression through an ARE-independent pathway. PLoS One.

[22] Iwasaki H, Takeuchi O, Teraguchi S, Matsushita K, Uehata T, Kuniyoshi K, Satoh T, Saitoh T, Matsushita M, Standley DM (2011). The IkappaB kinase complex regulates the stability of cytokine-encoding mRNA induced by TLR-IL-1R by controlling degradation of regnase-1. Nat Immunol.

[23] Tsubuki S, Saito Y, Tomioka M, Ito H, Kawashima S (1996). Differential inhibition of calpain and proteasome activities by peptidyl aldehydes of di-leucine and tri-leucine. J Biochem.

[24] Chen JJ, Huang WC, Chen CC (2005). Transcriptional regulation of cyclooxygenase-2 in response to proteasome inhibitors involves reactive oxygen species-mediated signaling pathway and recruitment of CCAAT/enhancer-binding protein delta and CREB-binding protein. Mol Biol Cell.

[25] Frezza M, Schmitt S, Dou QP (2011). Targeting the ubiquitin-proteasome pathway: an emerging concept in cancer therapy. Curr Top Med Chem.

[26] Sears C, Olesen J, Rubin D, Finley D, Maniatis T (1998). NF-kappa B p105 processing via the ubiquitin-proteasome pathway. J Biol Chem.

[27] Rape M, Jentsch S (2002). Taking a bite: proteasomal protein processing. Nat Cell Biol.

[28] Yao T, Ndoja A (2012). Regulation of gene expression by the ubiquitin-proteasome system. Semin Cell Dev Biol.

[29] Uhlen M, Oksvold P, Fagerberg L, Lundberg E, Jonasson K, Forsberg M, Zwahlen M, Kampf C, Wester K, Hober S (2010). Towards a knowledge-based Human Protein Atlas. Nat Biotechnol.

[30] Shibata T, Imaizumi T, Tamo W, Matsumiya T, Kumagai M, Cui XF, Yoshida H, Takaya S, Fukuda I, Satoh K (2002). Proteasome inhibitor MG-132 enhances the expression of interleukin-6 in human umbilical vein endothelial cells: involvement of MAP/ERK kinase. Immunol Cell Biol.

[31] Wu HM, Wen HC, Lin WW (2002). Proteasome inhibitors stimulate interleukin-8 expression via Ras and apoptosis signal-regulating kinase-dependent extracellular signal-related kinase and c-Jun N-terminal kinase activation. Am J Respir Cell Mol Biol.

[32] Wu WT, Chi KH, Ho FM, Tsao WC, Lin WW (2004). Proteasome inhibitors up-regulate haem oxygenase-1 gene expression: requirement of p38 MAPK (mitogen-activated protein kinase) activation but not of NF-kappaB (nuclear factor kappaB) inhibition. Biochem J.

[33] Kasza A, Wyrzykowska P, Horwacik I, Tymoszuk P, Mizgalska D, Palmer K, Rokita H, Sharrocks AD, Jura J (2010). Transcription factors Elk-1 and SRF are engaged in IL1-dependent regulation of ZC3H12A expression. BMC Mol Biol.

[34] Fiedler MA, Wernke-Dollries K, Stark JM (1998). Inhibition of TNF-alpha-induced NF-kappaB activation and IL-8 release in A549 cells with the proteasome inhibitor MG-132. Am J Respir Cell Mol Biol.

[35] Frevel MA, Bakheet T, Silva AM, Hissong JG, Khabar KS, Williams BR (2003). p38 Mitogen-activated protein kinase-dependent and -independent signaling of mRNA stability of AU-rich element-containing transcripts. Mol Cell Biol.

[36] Shimizu S, Kadowaki M, Yoshioka H, Kambe A, Watanabe T, Kinyamu HK, Eling TE (2013). Proteasome inhibitor MG132 induces NAG-1/GDF15 expression through the p38 MAPK pathway in glioblastoma cells. Biochem Biophys Res Commun.

[37] Brook M, Tchen CR, Santalucia T, McIlrath J, Arthur JS, Saklatvala J, Clark AR (2006). Posttranslational regulation of tristetraprolin subcellular localization and protein stability by p38 mitogen-activated protein kinase and extracellular signal-regulated kinase pathways. Mol Cell Biol.

[38] Younce CW, Wang K, Kolattukudy PE (2010). Hyperglycaemia-induced cardiomyocyte death is mediated via MCP-1 production and induction of a novel zinc-finger protein MCPIP. Cardiovasc Res.

[39] Niu J, Wang K, Graham S, Azfer A, Kolattukudy PE (2011). MCP-1-induced protein attenuates endotoxin-induced myocardial dysfunction by suppressing cardiac NF-κB activation via inhibition of IκB kinase activation. J Mol Cell Cardiol.

[40] Nasuhara Y, Adcock IM, Catley M, Barnes PJ, Newton R (1999). Differential IkappaB kinase activation and IkappaBalpha degradation by interleukin-1beta and tumor necrosis factor-alpha in human U937 monocytic cells. Evidence for additional regulatory steps in kappaB-dependent transcription. J Biol Chem.

[41] Calvaruso G, Giuliano M, Portanova P, De Blasio A, Vento R, Tesoriere G (2006). Bortezomib induces in HepG2 cells IkappaBalpha degradation mediated by caspase-8. Mol Cell Biochem.

[42] Li C, Chen S, Yue P, Deng X, Lonial S, Khuri FR, Sun SY (2010). Proteasome inhibitor PS-341 (bortezomib) induces calpain-dependent IkappaB(alpha) degradation. J Biol Chem.

[43] Kaltschmidt B, Kaltschmidt C, Hehner SP, Droge W, Schmitz ML (1999). Repression of NF-kappaB impairs HeLa cell proliferation by functional interference with cell cycle checkpoint regulators. Oncogene.

[44] Schneider CA, Rasband WS, Eliceiri KW (2012). NIH Image to ImageJ: 25 years of image analysis. Nat Methods.

